# Tea and Coffee as the Cause of Sick-Headache

**Published:** 1840-10

**Authors:** 


### TEA AND COFFEE, AS THE CAUSE OF SICK-HEADACHE.

Dr. Brooks, of Harrisonville, Mo., demolishes the theory of Dr. Burbell, noticed in our July No., that the use of tea and coffee is the source of the accumulating anguish which flows from this disease. He proceeds on this wise:

"I was brought up in Kentucky in early times when it was not easy to procure tea and coffee, and when, consequently, they were little used. Those who lived most luxuriously drank coffee once a week, but then it was not made strong, as now, but was frequently mixed with parched rye or wheat. In those days, I had around me a connexion of about a thousand souls, all of whom, except one, were more or less addicted to this complaint. Since that time, my family connexion has much increased, and from all I can learn sick-headache has diminished amongst them, notwithstanding that, like most other people, they have fallen into the habit of drinking tea and coffee, many of them to excess. I do not wish to be understood as expressing a belief, that these beverages are remedies for the disease, but only that they are not the most common cause of it, and that total abstinence from them will not relieve every case. I have had a fair opportunity of knowing something about this matter—I am upwards of fifty-two years of age, and have been engaged in the practice of medicine for nearly thirty years.

"I wish to make an enquiry about a change which sometimes takes place in children after birth, commonly called *double lip.* I have seen no mention of it in any medical work, nor have I ever heard a medical man speak of the infirmity. Within the last three and twenty years I have met with it in thirty-one children, who were born without it, which I believe is always the case. The deformity commences generally a little to one side of the lip, and gradually the inside protrudes downwards. I believe it is confined to the upper lip. What is the cause of this affection? Can it be prevented, or cured when it has become confirmed." Y.

